# A rational treatment of Mendelian genetics

**DOI:** 10.1186/1742-4682-1-6

**Published:** 2004-08-31

**Authors:** John W Porteous

**Affiliations:** 1 Department of Molecular and Cell Biology, Institute of Medical Sciences, University of Aberdeen, Foresterhill, Aberdeen AB25 2ZD, Scotland, UK

## Abstract

**Background:**

The key to a rational treatment of elementary Mendelian genetics, specifically to an understanding of the origin of dominant and recessive traits, lies in the facts that: (1) alleles of genes encode polypeptides; (2) most polypeptides are catalysts, i.e. enzymes or translocators; (3) the molecular components of all traits in all cells are the products of systems of enzymes, i.e. of fluxing metabolic pathways; (4) any flux to the molecular components of a trait responds non-linearly (non-additively) to graded mutations in the activity of any one of the enzymes at a catalytic locus in a metabolic system; (5) as the flux responds to graded changes in the activity of an enzyme, the concentrations of the molecular components of a trait also change.

**Conclusions:**

It is then possible to account rationally, and without misrepresenting Mendel, for: the origin of dominant and recessive traits; the occurrence of Mendel's 3(dominant):1(recessive) trait ratio; deviations from this ratio; the absence of dominant and recessive traits in some circumstances, the occurrence of a blending of traits in others; the frequent occurrence of pleiotropy and epistasis.

## 1. Background

**Figure 2:**
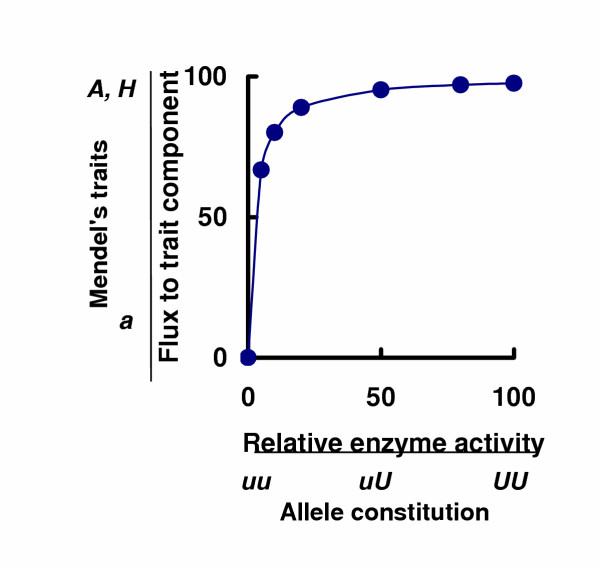
Accounting for Mendel's observation of a 3(dominant):1(recessive) trait ratio in his F2 populations of plants. Mendel's notations for a dominant trait, a hybrid and a recessive trait were (*A*), (*Aa*) and (*a*) respectively. For reasons given in the preceding paper [1], a hybrid trait is represented in Figure 2 by (*H*). The molecular components of all traits are synthesised by a metabolic pathway. When the activity of any one enzyme in a metabolic pathway is changed in discrete steps, the flux to a trait component responds in non-linear (non-additive) fashion [3]. If the flux response is quasi-hyperbolic, as shown here, the hybrid trait (*H*) will be indistinguishable from the trait (*A*) expressed in the wild-type cell or organism, even when the enzyme activity in the hybrid (*H*) has been reduced to 50% of the wild-type activity. Trait (*a*), will be distinguishable from both traits (*A*) and (*H*) only if the enzyme activity is further reduced to a sufficient extent. Under these circumstances the trait series (*A *+ 2*H *+ *a*) becomes (3*A *+ *a*); Mendel's 3(dominant):1(recessive) trait ratio is accounted for without introducing arbitrary and inconsistent arguments [1].

The currently favoured explanation for the origin of Mendel's dominant and recessive traits is untenable [1]. The primary error in this current attempted explanation is the assumption that there is a direct, proportional, relationship in a diploid cell between a series of allegedly dominant and recessive alleles written as (*AA *+ 2*Aa *+ *aa*) and the dominant, hybrid and recessive traits written as (*AA *+ 2*Aa *+ *aa*). This assumption (Figure 2, in reference [1]) incorporates four fundamental faults:

(i) A failure to distinguish between the parameters and the variables of any system of interacting components, specifically between the determinants (alleles in modern terminology) and what is determined (the form of the trait or characteristic expressed in a cell or organism). Thus, because Mendel defined the terms dominant and recessive for *traits *or *characters*, it was illegitimate (and illogical) to call alleles dominant or recessive, and to represent them by the same letters used by Mendel to represent traits [1].

(ii) A trait series written as (*AA *+ 2*Aa *+ *aa*) suggests, incorrectly, that dominant and recessive traits comprise two aliquots, (*A + A*) or (*a *+ *a*), of dominance or recessivity.

(iii) A failure to take account of the long established fact that the first non-nucleotide product of the expression of an allele is a polypeptide and that most polypeptides are enzymes or membrane-located translocators.

(iv) A failure to note that the components of all tangible traits comprised the molecular products of metabolic pathways, i.e., the products of sequences of enzyme-catalysed reactions.

Correction of the first two of these four faults has already been achieved (section 4 in reference [1]) by writing an allele series as (*UU *+ 2*Uu *+ *uu*) and the corresponding trait series as (*A *+ 2*H *+ *a*). In these statements (*U*) and (*u*) are normal and mutant (not dominant and recessive) alleles respectively. Mendel's notation (*A*) and (*a*) is used to represent dominant and recessive traits but (*H*) replaces Mendel's implausible notation (*Aa*) for a hybrid class of trait [1]. Mutations at another gene locus, in the same or a different cell, will be written as (*WW *+ *Ww *+ *ww*); the corresponding trait series will appear as (*B *+ 2*H *+ *b*). Mendel's notation (*Aa*) for a hybrid trait will be used in this article only when referring directly to Mendel's paper [2].

## 2. A rational explanation of Mendel's observations

Our stated task was to explain logically how an allele series (*UU *+ 2*Uu *+ *uu*) is expressed as a series of qualitatively distinguishable F2 traits (*A *+ 2*H *+ *a*) when F1 hybrids (*H*) are allowed to self-fertilise [1]. This is very simply achieved by correcting faults (iii) and (iv) in four successive steps (sections 2.1–2.4) based on a paper published 23 years ago [3]. A fifth step (section 2.5) allows us to go beyond that paper to explain how the trait ratio 3(dominant):1(recessive) sometimes occurs and sometimes does not. A sixth step (section 2.6), consistent with the earlier ones, explains why dominance and recessivity are not always observed. Section 2.7 validates an earlier section. Section 2.8 accounts for some aspects not dealt with in textbooks and reviews of genetics.

The treatment in this section 2 is extended in section 3 to account for quantitatively different traits, in section 4 to illustrate some implications of the present treatment, and in section 5 to account for pleiotropy and epistasis. Section 6 defines the conditions that must be met if a rational account is to be given for the occurrence of dominant and recessive traits.

### 2.1. A generalised metabolic system

If: the first non-nucleotide product of expression of an allele is a polypeptide and most polypeptides are enzymes [3,4], it follows that most mutations at *any one *gene locus will result in the formation of a mutant enzyme at a catalytic locus in a metabolic pathway. This is true even if the functioning enzyme is composed of more than one polypeptide, each specified by different genes. It then follows that we need to ask how the concentration of a normal molecular component of a trait will be affected by a mutation of *any one *enzyme *within a metabolic system*. In short, a systemic approach, outlined below, is obligatory.

This is the key to an understanding of the origin of dominant and recessive traits, as first pointed out in the following two sentences: "When as geneticists, we consider substitutions of alleles at a locus, as biochemists, we consider alterations in catalytic parameters at an enzyme step. - -. The effect on the phenotype of altering the genetic specification of a single enzyme - - - is unpredictable from a knowledge of events at that step alone and must involve the response of the system to alterations of single enzymes *when they are embedded in the matrix of all other enzymes*." ([3]; p.641).

### 2.2 Metabolic systems and steady states

**Figure 1:**
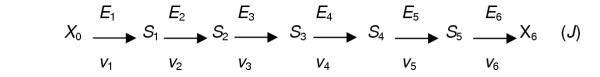
A segment of a model metabolic pathway. This diagram shows those features, discussed in the text, that permit a systemic analysis of the response of any variable of a metabolic system (e.g. a flux *J *or the concentration of any intracellular metabolite S) to changes in any one parameter of the system (e.g. an enzyme activity). Each *S *is an intracellular metabolite; each *X *is an extracellular metabolite. In a diploid cell, every *E *stands for a pair of enzymes (allozymes), each specified by one of the two alleles at a gene locus. Each *E *is then a locus of catalytic activity within a system of enzymes; each *v *stands for the individual reaction rates catalysed jointly by a pair of allozymes in a diploid cell. Either or both allozymes at such a locus may be mutated.

Metabolic processes are facilitated by a succession of catalysed steps; i.e. by enzyme-catalysed transformations of substrates to products or by carrier-catalysed translocation of metabolites across membranes. Because enzymes and membrane-located carriers (or porters) are saturable catalysts that exhibit similar kinetics it is convenient in this article to refer only to enzymes and to represent both kinds of catalysts by the letter *E*. Any segment of a sequence of enzyme-catalysed reactions can then be written as shown in Figure 1.

There are ten important features of any such system.

(1) Each enzyme, *E*_1 _to *E*_6_, is embedded within a metabolic pathway, i.e. within a system of enzymes.

(2) All components of this system except the *external *metabolites *X*_0 _and *X*_6 _are enclosed by a membrane.

(3) *E*_1 _and *E*_6 _may then represent membrane-located enzymes or translocators.

(4)*X*_0 _and *X*_6 _interact with only one enzyme, whereas each internal metabolite (*S*_1_, *S*_2_, *S*_3_, *S*_4_, *S*_5_) interacts with two flanking enzymes.

(5) In a haploid cell there will be one specimen of an enzyme molecule (*E*) at each catalytic locus. In a diploid cell there will be two specimens of enzyme molecules (two allozymes) at each catalytic locus: one specified by the maternal allele, the other by the paternal allele, at the corresponding gene locus or loci. The effective catalytic activity at each metabolic locus in a diploid will be, in the simplest case, the sum of the two individual activities. It is the single effective enzyme activity (*v*) at each catalytic locus that concerns us here, irrespective of whether the cell is haploid, diploid or polyploid.

(6) The catalytic activity (*v*) at any one metabolic locus can be left at its current value or changed to and *maintained at *a new value by the experimentalist, e.g. by suitable genetic manipulation of an allele. Each allele in these circumstances is therefore an *internal parameter *of the system; it is accessible to modification by the direct and sole intervention of the experimentalist [1].

(7) Because *X*_0 _and *X*_6 _are external to the system in Figure 1, their concentrations can be fixed, and maintained at a chosen value, by the direct intervention of the experimentalist; they are *external parameters *of the metabolic system.

(8) In contrast to *X*_0 _and *X*_6_, the concentrations of metabolites *S*_1 _to *S*_5 _within the system cannot be fixed and maintained at any desired value solely by the direct intervention of the experimentalist. The concentrations of *S*_1 _to *S*_5 _are *internal variables *of the system. (If a fixed amount of any one of these metabolites were to be injected through the membrane into the system, continued metabolism would ensure that the new intracellular metabolite concentration could not be maintained).

(9) By the same arguments, each reaction rate (*v*) and the flux (*J*) through the system are also variables of the system.

(10) The magnitude of each variable of the system is determined at all times by the magnitudes of all the parameters of the system and of its immediate environment. The variables comprise the *concentrations *(*s*_1_, *s*_2_, *s*_3_, *s*_4_,*s*_5_) of the intracellular metabolites shown in Figure 1 and any other intracellular metabolites; the individual reaction rates *v*_1_, *v*_2_, *v*_3_, *v*_4_, *v*_5_, *v*_6_; and the flux *J *through this system of enzyme-catalysed steps.

It follows that, provided we maintain the concentrations of *X*_0 _and *X*_6 _constant, the system depicted (Figure 1) will, in time, come to a steady state such that:

*v*_1 _= *v*_2 _= *v*_3 _= *v*_4 _= *v*_5 _= *v*_6 _= *J *(the flux through this system).

At the same time the concentration of each intracellular metabolite *S*_1 _to *S*_5 _will settle to an *individual *steady value.

### 2.3. The response of the system variables to a change in any one system parameter

In a metabolic system, the product of any one enzyme-catalysed reaction is the substrate for the immediately adjacent downstream enzyme (Figure 1). If, for any reason, the concentration of the common intermediate metabolite of two adjacent enzymes is changed (for example by mutation of one of the two adjacent enzymes), the concentration of the other adjacent enzyme will not change but its activity will change in accordance with the known response of an enzyme activity (at constant enzyme concentration) to a change in the concentration of its substrate or product. In other words, no matter how complicated that system may be, the *activity *of any one enzyme depends, at all times, on the activity of the adjacent enzyme; and this is true for every pair of adjacent enzymes throughout the system (up to the point in the system where a terminal product is formed).

[This last statement is obviously still true for the system in Figure 1 if we omit the words in parentheses but only because the extracellular product *X*_6 _is a terminal product. *X*_6 _is not an intermediate metabolite, flanked by *two *adjacent enzymes; it is not a substrate that is further metabolised by the system depicted. There are instances where an intracellular terminal product is formed. We must therefore add the words in parentheses if the statement is to apply generally].

A finite change (by mutation) in any one allele at a locus will change the activity (*v*) of one enzyme at the corresponding metabolic locus; but, for reasons just stated in the first paragraph of this section 2.3, the activity (*v*) of each of the other enzymes will alter, the flux (*J*) will change, and the *concentrations *of all the metabolites (*S*_1_-*S*_5_) will also change, some more than others, until the system settles to a new steady state.

Thus, finite changes in the magnitude of *any one *of the internal or external *parameters *of the system will shift the original values of *all *the *variables *of the system to a new set of steady-state values. But, providing the external parameters *X*_0 _and *X*_6 _are kept constant, we can be sure that a change in any one selected internal parameter (an allele or an enzyme) would be the sole cause of any changes in the system variables. In short, we are obliged to adopt a whole-system (a systemic) approach if we want to understand how the flux to a trait component responds to a change in any one internal or external parameter of the system, no matter how that change in a parameter value is brought about. We are here concerned with changes in any one *internal *parameter such as a *mutation *in one or both alleles of a diploid cell.

Suppose the activity of any one of the enzymes *E*_1 _to *E*_6 _in Figure 1 were to be changed stepwise (e.g. by a series of mutations of one or both alleles at a locus in a diploid) so that the residual activity of the enzyme was decreased in successive steps to, say, 75%, 60%, 45%, 25%, 0% of its initial activity. How would the flux (flow) through the whole series of enzymes vary; i.e. how would the flux (to a trait component) respond, and how would the concentration of that molecular component of a trait respond, when any one enzyme activity was changed by mutation in a series of finite steps?

It was shown, by experiment, that graded changes in the activity of any one of four different enzymes in the arginine pathway resulted in a non-linear (quasi-hyperbolic) response of the flux to arginine in constructed heterokaryons of *Neurospora crassa *([3], Figures 1a,1b,1c,1d). Similar non-linear (non-additive) flux responses were observed when a series of mutations occurred in a single enzyme in four other metabolic pathways in four different diploid or polyploid systems ([3], Figures 1e,1f,1g,1h). Similar flux responses were observed during genetic down-modulation of any one of five enzymes involved in tryptophan synthesis in *Saccharomyces cerevisiae *[5]. The same quasi-hyperbolic response of a defined flux to a series of graded changes in one enzyme activity was observed in a haploid cell [6]. We can therefore dismiss the possibility that these non-linear responses (of a flux-to-a-trait-component) were restricted to the systems investigated by Kacser and Burns [3] or were in some way related to the ploidy of the cells and organisms they studied.

On the contrary, the various flux responses are a fundamental *biochemical property *of the fluxing metabolic system. It does not matter how the graded changes in activity of any one enzyme are brought about. Mutation is one way but not the only one. Graded replacement of a defective gene that expressed the chloride translocator in the cystic fibrosis mouse produced continuously non-additive responses of various functions associated with chloride transport, including the duration of the survival of the mouse [7]. Induced synthesis of graded concentrations of a single membrane-located enzyme resulted in continuously non-linear changes in growth rate, glucose oxidation, the uptake and phosphorylation of α-methyl glucose by *Escherichia coli *cells [8].

Stepwise decreases in cytochrome c oxidase activity (by titrating rat muscle mitochondria with an enzyme-specific inhibitor) had little effect on respiration until the enzyme activity was decreased to about 25% of normal; further decreases in this one enzyme activity caused a precipitous, continuously non-linear, decrease in mitochondrial respiration [9]. Other examples of non-linear (non-additive) responses of a defined flux to a change in activity of one enzyme in a metabolising system have been recorded [10], [[11], Figures 6.2,6.3,6.4,6.6.6.7,6.8]. The results of these various "genetic" and "biochemical" experiments illustrate the generality of the statement by Kacser and Burns [3] quoted in section 2.1 of this article.

**Figure 6:**
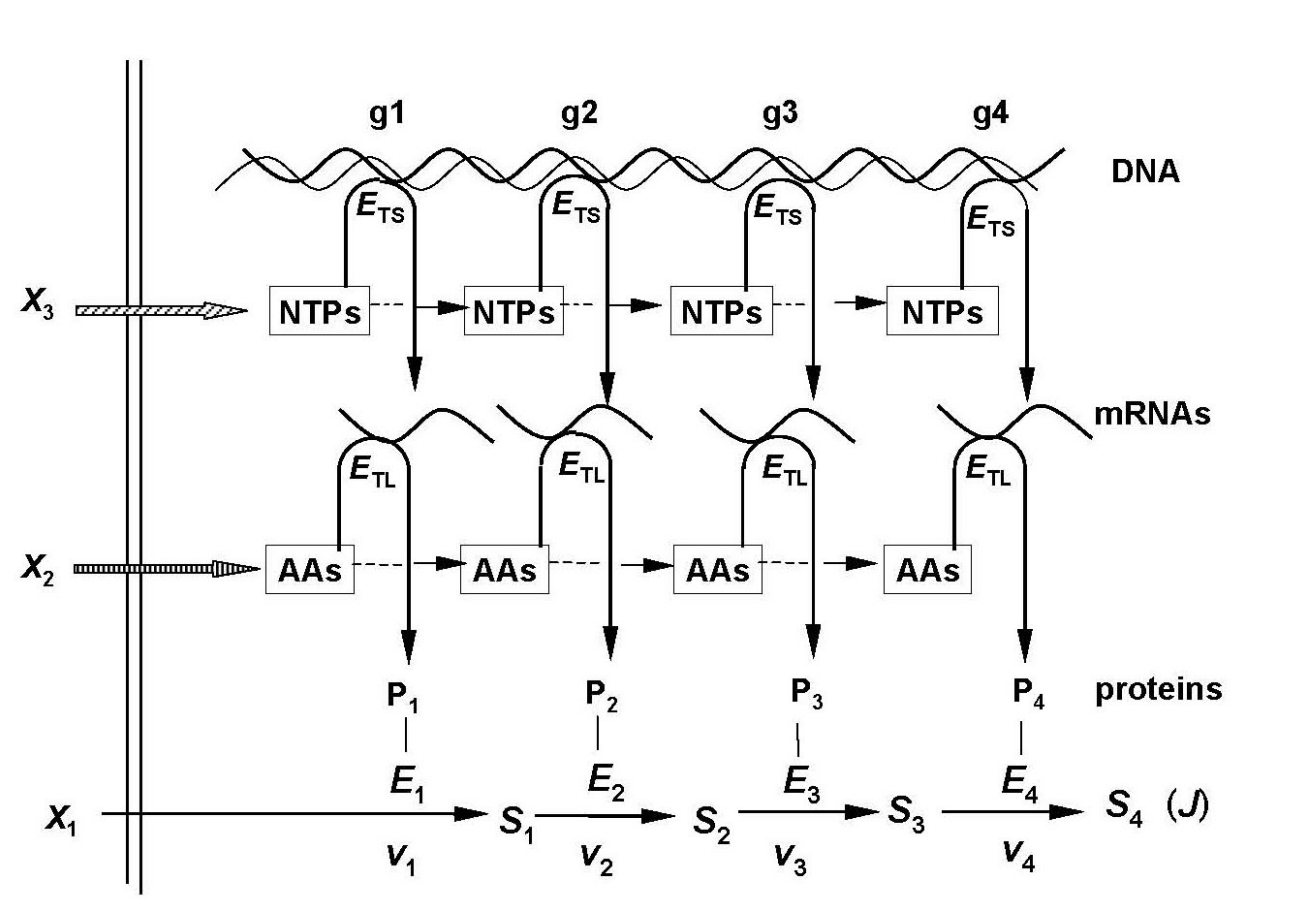
Biochemistry and genetics merged thirty years ago. The symbol  indicates the catalysed translocation of an extracellular substrate or substrates (*X*_3_) and the subsequent intracellular catalysed transformations, including scavenging pathways, that form nucleoside triphosphate (NTP) precursors for the transcription process. Similarly,  indicates the catalysed translocation of the extracellular substrates (*X*_2_) and the subsequent synthesis from (*X*_2_), and other intracellular substrates, of the amino acid (AA) precursors for the translation process. The enzymes subsumed as *E*_Ts _and *E*_Tl _are involved in the final stages of the expression (transcription and translation) of genes g1, g2, g3, g4 - - etc as polypeptides (P_1_, P_2_, P_3_, P_4 _- - etc). In diploid cells a pair of proteins will be synthesised from each pair of alleles at a gene locus. Those pairs of polypeptides (proteins) that are catalytically active in a diploid cell are represented by the single symbols *E*_1_, *E*_2_, *E*_3_, *E*_4 _- - - etc in this Figure 6. Further details are given in Section 5.5.

### 2.4. A rational explanation for the origin of dominant and recessive traits

How did the observations of non-linear responses of individual fluxes to graded changes in any one enzyme activity lead to a rational explanation for the origin of Mendel's dominant and recessive trait classes [2]? For reasons already given, we cannot arrive at the answers to this question by relying on the illogical and illegitimate idea that alleles are themselves dominant or recessive. Such entities have never existed and do not now exist. Alleles can only be normal or abnormal (i.e. normal or mutant). If the ploidy of the cell cannot explain the non-additive response of a flux to mutations in an allele, it is equally certain that naming alleles as dominant or recessive will not provide the explanation [1]. We need to focus attention on the universally observed non-linear (often quasi-hyperbolic) responses of the flux-to-a-trait-component (and the concomitant change in concentration of that component) when the activity of any one enzyme, within a metabolic system of enzymes, is changed (decreased or increased), in stages, by any means available (including down-modulation by mutation and up-modulation by increasing the gene dose).

In this Section 2.4, and in Sections 2.5–2.7, consideration of the role of allele pairs (*uu*,*uU*,*UU*) in determining the outcome of mutations or changes in gene dose is set aside; this role will be considered in Section 2.8. For the moment, attention is focussed on what can be learned from the non-linear response of a flux – to the molecular component(s) of a trait – when the activity of one enzyme in a metabolic system is changed in graded steps by mutation or by changes in gene dose. Figures 1a,1b,1c,1d in Reference [3] showed that the flux to the normal trait component (arginine), and thus the concentration of arginine, was not significantly diminished before any one of four enzyme activities was decreased by more than 50%. In Figures 1b,1d the enzyme activity was decreased to about 15% of normal activity in *Neurospora crassa *before any significant diminution in the flux to arginine (and in the concentration of arginine) was detectable [3]; any further diminution of either enzyme activity caused a continuous but precipitous fall in the production of this trait component. Similar characteristics were displayed by a diploid (Figure 1h in Reference [3]). Figure 2 represents these observations. Flux response plots with these characteristics are quasi-hyperbolic and asymmetric in the sense that, over low ranges of enzyme activity, the flux (and the metabolite concentrations in that fluxing pathway) respond markedly to small increases or decreases in enzyme activity; on the other hand, over high ranges of enzyme activity, substantial changes in activity have a small, if any, effect on the flux to a trait component and on the concentrations of the molecular components of a defined trait. A change in any "Flux-to-trait-component" implies a change in the concentrations of those metabolic products that typify a defined trait.

It was shown that a dominant trait (*A*) corresponded to the normal (100%) activity of the enzyme that was subsequently mutated to give lower activities [3]; i.e., the plotting co-ordinate (wild-type enzyme activity *versus *trait *A*) defined the terminus of the asymptote of the flux response plot depicted in Figure 2. A hybrid (*H*) must then correspond to *any *point on the asymptote of Figure 2 that would not allow us (and would not have allowed Mendel) to distinguish a F1 hybrid (*H*) from its parent that displayed a dominant trait (*A*). A recessive (*a*) must then correspond to *any *point on the steeply falling part of the flux-response plot (Figure 2) that would allow us (or would have allowed Mendel) to *distinguish *the dominant trait (*A*) and the hybrid (*H*) from the recessive trait (*a*), e.g. dominant trait red flowers and hybrid red flowers from the recessive trait white flowers [1]. Note especially that a recessive trait would not *necessarily *correspond to zero flux (a complete metabolic block and a complete absence of the normal, downstream, metabolic products) in Figure 2.

The paper by Kacser and Burns [3] thus explains, for the first time in 115 years, how recessive *traits *arise from a sufficient decrease, by mutation, in one enzyme activity when that enzyme is embedded in a metabolic system. The explanation depends on recognising that when graded changes occur by mutation (in one, both or all of the allozymes at *any one *metabolic locus in biochemical pathways) there will be a non-linear response of the flux to the molecular component(s) of a defined trait; and concurrently a non-linear response of the concentrations of the normal molecular components of a trait (section 2.3).

Section 2.9 in reference [1] showed that it was difficult to understand how Mendel's recessive traits (*a*) were displayed in 1/4 of his F2 population of plants (*A *+ 2*Aa *+ *a*) when these same recessive traits were not displayed in Mendel's hybrids (*Aa*). We have replaced Mendel's implausible idea that his F1 hybrids (*Aa*) displayed only trait (*A*). We have substituted the plausible idea – based on experimental evidence [3] – that, under certain conditions, the F1 hybrid trait (*H*) is *indistinguishable *from trait (*A*). In the treatment advocated here, there is no problem in understanding how 1/4 of the individual plants in the F2 population of genetically related plants (*A *+ 2*H *+ *a*) displayed the recessive trait (*a*). We can now also see why Mendel emphasised the need to study crosses between parental plants that displayed readily distinguishable trait forms, e.g. red flowers (*A*) in one parent and white flowers (*a*) in the other [1]. Figure 2 shows that this distinction would be possible only if the activity of one enzyme in the dominant trait plant was sufficiently diminished in the recessive trait plant.

Note too that *trait *dominance and *trait *recessivity are not independent phenomena (nor are they opposite, one to the other). We cannot define a dominant trait except as an alternative to a recessive trait; both traits must be observable before we can identify either of them. The statements in these last two sentences were obvious in Mendel's original paper [2] but they have been inexplicably overlooked by many later authors.

### 2.5. Mendel's 3(dominant):1(recessive) trait ratio occurs sometimes, not always

Does this explanation for the *origin *of dominant and recessive traits also account for the occurrence of Mendel's 3(dominant):1(recessive) trait ratio? The answer is yes. Does it also explain why this ratio is not always observed? The answer is again, yes (although the original authors [3] did not pose or answer these two questions).

If the flux response plot is *sufficiently asymmetric *(approaches a hyperbolic plot, as in Figure 2), the concentration of molecular components of a defined trait will not be measurably different (when the activity of one enzyme is decreased by, say, 50%) from the concentrations of those same molecular components when the enzyme activity was 100%.

If the trait displayed by the hybrid (*H*) is indistinguishable from the trait (*A*), as in Figure 2, the trait distribution in the F2 population (*A *+ 2*H *+ *a*) becomes 3(*A*) + (*a*); i.e. the trait ratio in this population will be 3(dominant):1(recessive). This explanation for the occurrence of the 3:1 trait ratio in Mendel's, or any other F2 population of cells or organisms, depends entirely on an experimentally observed, sufficiently asymmetric, response of the flux (to the molecular components of defined trait) when changes occur in enzyme activity at any one metabolic locus in a fluxing biochemical pathway (Figure 1). It does not depend on the naïve and illegitimate assumption that alleles are either dominant or recessive (Sections 3.2, 3.3, 4 in Reference [1]).

**Figure 3:**
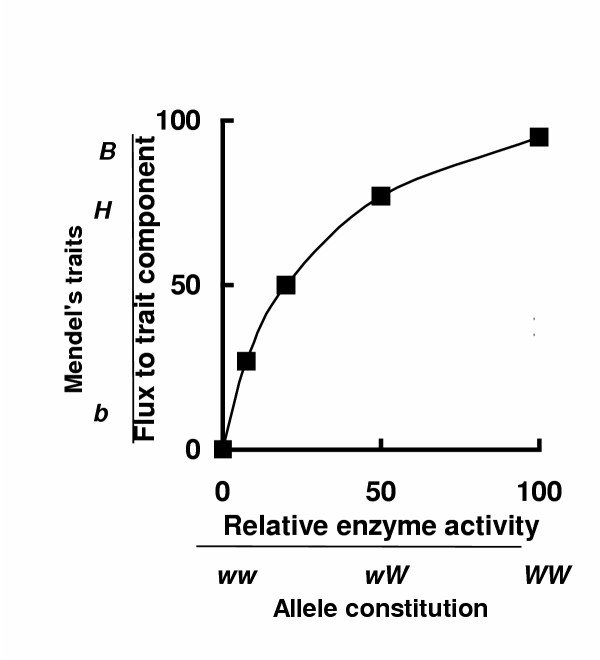
Mendel's 3(dominant):1(recessive) trait ratio does not always occur. Mendel's notation for a dominant trait, a hybrid and a recessive trait were (*B*), (*Bb*) and (*b*) respectively. For reasons given in the preceding paper [1], the hybrid is represented in Figure 3 by (*H*). When graded changes are made in any one enzyme in a metabolic pathway the response of the flux through that pathway is always non-linear (non-additive) but not always quasi-hyperbolic (Figure 2). Consequently when the enzyme activity at one metabolic locus is decreased in the heterozygote to (say) 50% of wild-type, the trait displayed by the hybrid (*H*) is now distinguishable from the trait (*B*) displayed by the wild type cell or organism and from the trait (*b*) displayed by the homozygously mutant cell or organism. Mendel's 3(dominant):1(recessive trait ratio will not be observed. The explanation is consistent with the explanation for the observation of the 3:1 trait ratio in Figure 2 and achieves what the currently favoured explanation of Mendel's observations cannot achieve [1].

Figure 2 illustrates one of a family of regularly non-linear (non-additive) response plots which exhibit various degrees of asymmetry [3]. Is the flux response always sufficiently asymmetric for the 3:1 trait ratio to be observed? It is not. A flux response was observed in one particular (diploid) metabolic system (Reference [3], Figure 1f) that was still clearly non-linear (non-additive) but not as asymmetric as that shown in Figure 2. As in Figure 2, so in Figure 3, a recessive trait (*b*) can be clearly distinguished from the dominant trait (*B*) because the concentrations of the molecular components of this trait were sufficiently different when one enzyme activity in the metabolic system is decreased to a sufficient extent. The trait displayed by the hybrid (*H*) is now *distinguishable *(rather than indistinguishable) from the dominant trait (*B*) expressed in a genetically related normal cell or organism when, as in Figure 2, the enzyme activity is decreased to an arbitrarily chosen 50% of the normal activity. The 3(dominant):1(recessive) trait ratio will not then be observed (Figure 2). A blend of traits (*B*) and (*b*) is possible in the hybrid (*H*), for example when traits (*B*) and (*b*) are distinguished by colour differences.

### 2.6. Dominant and recessive traits are not always observed

It is well known that dominance and recessivity are not universally observed. Are they therefore of no significance? Some authors have been tempted to think so. Their view is understandable because, before the work of Kacser and Burns [3], we lacked any credible explanation for the occurrence of dominant and recessive traits.

Can we now see why dominance and recessivity are not always observed? The answer is again, yes. Examination of Figure 2 and Figure 3 shows that it will be possible to observe dominant *and *recessive traits in genetically related organisms only when the enzyme activity at a metabolic locus is decreased from 100% to an activity approaching, but not necessarily reaching, 0% activity.

When the response plot is of the kind shown in Figure 2, it would be possible to decrease the expressed enzyme activity at a metabolic locus by at least 75%, perhaps by 85%, without eliciting any detectable change in trait from that displayed by the wild-type or normal organism. In other words some mutations will not, apparently, display Mendelian dominance and recessivity (dominant and recessive *traits*).

Only if the effective enzyme activity is decreased by at least 95% in this instance (Figure 2), would clear dominance and recessivity be noted. This is an extreme case; Figure 3 illustrates the other extreme. Between these extremes, various degrees of asymmetry of flux response plots may be observed (Figure 1 in Reference [3]). Nevertheless, unless: (i) the change in enzyme activity is measured, (ii) it is realised that there is a non-additive relationship between a change in any one enzyme activity at a metabolic locus and a change in expressed trait, and (iii) the shape of the flux response plot (Figure 2, Figure 3) is revealed by plotting, it is simply not possible to state that the system under investigation does or does not display Mendelian dominance and recessivity. Terms such as semi-dominance merely indicate that the flux response plot is not quite asymmetric enough to be sure that a 50% reduction in enzyme activity produces a trait that is indistinguishable from the dominant trait.

### 2.7. Is the Kacser & Burns treatment universally applicable?

The change in the *concentrations *a normal metabolites has been treated in the present article as the source of a change in trait. This accords with the treatment in Figure 1 of reference [3]. Allowance should, however, be made for the possibility that the change in concentration of a metabolite is, in reality, a change in the concentration of a "signalling" metabolite (e.g. an allosteric activator or inhibitor of another enzyme in the pathway that generated the "signalling" metabolite, or in another pathway). Such mechanisms merely shift the cause of the change in metabolite concentration to another part of the matrix of intracellular metabolic pathways. In other words, the Kacser and Burns approach remains a valid explanation for the origin of dominant and recessive traits.

### 2.8. Accounting for all the plotting points in Figures 2 and 3

In Figure 2, the relative enzyme activities (100, 50, 0) would be expressed from the series of allele pairs *UU*, *Uu*, *uu *in a diploid cell (Section 1) only if the mutant allele (*u*) was expressed as a catalytically inactive polypeptide. The same considerations apply to the relative enzyme activities expressed from the allele pairs *WW*, *Ww*, *ww *in Figure 3.

It is obvious that the continuously non-linear response plots (Figures 2, 3; and References [3-10]]) could not be constructed if these three allele pairs were the only ones available to express a corresponding series of enzyme activities. Figure 1 in Reference [3] showed that more than three distinct enzyme activities were observed in experimental practice in any one system. It is easy to see how relative enzyme activities other than 0, 50, 100 could be observed in a polyploid or heterokaryon (Figure 1a,1b,1c,1d,1e in Reference [3]). To account for the occurrence in a diploid of relative enzyme activities in addition to those taking values of 0, 50, 100 (in Figures 2 and 3, and in Figures 1f,1g,1h of Reference [3]), we need to allow for allele pairs in addition to the three (*UU*, *Uu*, *uu *or *WW*, *Ww*, *ww*) in which the mutant alleles (*u *or *w*) express a catalytically inactive polypeptide.

The restriction to just three allele pairs in a diploid may be traced to Sutton [1]. He wrote Mendel's F2 trait series (*A *+ 2*Aa *+ *a*), incorrectly, as (*AA *+ 2*Aa *+ *aa*) and the number of distinguishable chromosome pairs as (*AA *+ 2*Aa *+ *aa*), so establishing a false one-for-one relationship between pairs of chromosomes (*AA *or *aa*) and dominant or recessive traits (*AA *or *aa*). Sutton's notation for chromosome pairs was later transferred to allele pairs. In this article, dominant and recessive traits are represented, as Mendel did, by (*A*) and (*a*) respectively; alleles have been represented by different letters (e.g. *UU*, *Uu*, *uu*) in order to distinguish alleles (parameters) from traits (variables). We should allow for the situation where (*U*^†^) is a mutant of (*U*) that would express an allozyme activity lower than that expressed from (*U*) but not so low as that expressed from (*u*); and where (u*) would be a mutant of (*U*) that expresses an allozyme activity greater than that expressed by (*u*) = 0 in the traditional treatment but not so great as to merit the notation (*U*). The outcome of different hypothetical crosses that involve different mutations of one both alleles at a given locus in genetically related diploid parents would then be as follows:

(1) Repeated crosses (*Uu *× *Uu*) would give, on average, the allele series (*UU *+ 2*Uu *+ *uu*) thus permitting expression of no more than three distinctive enzyme activities at the corresponding metabolic locus.

(2) The cross (Uu* × *Uu*) would give the allele series (*UU *+ *Uu *+ Uu* + uu*) in which two of the allele pairs differ from those in the progeny of the first cross; and in which three different heterozygotes are formed.

(3) The cross (*U*^†^*u *× *Uu*) would give the allele series (*UU*^† ^+ *Uu *+ *U*^†^*u *+ *uu*) in which only one allele pair in the progeny populations is identical with one of the allele pairs in the progeny from the second cross.

(4) The cross (*UU*^† ^× *Uu*) would give, on average, the allele series (*UU*^†^+ *UU *+ *Uu *+ *U*^†^*u*) which has only two allele pairs in common with the progeny of the third of these crosses of genetically related parents.

(5) The cross (*U*^†^*u *× Uu*) would give, on average, the allele series *(UU*^†^*+ U*^†^u* + Uu + uu*).

In the second and fourth crosses it was assumed that the two heterozygous parents did possess exactly the same normal allele (*U*) at this particular locus so, among their progeny, the allele pair (*UU*) occurred. Analogously, among the progeny from the third cross, the allele pair (*uu*) occurred. But, importantly, in each of crosses (2), (3) and (4) three different heterozygotes occurred in each progeny population (a heterozygote is defined in a diploid by the occurrence of allele pairs other than those represented here by *UU *or *uu*). The allele pairs in the heterozygotes in any one progeny population of these crosses (2), (3) and (4) are not all identical with those in the progeny of another of these crosses. The parents in the fifth cross did not share an identical allele; no two alleles of a pair are then identical in the progeny. The allele pair (*Uu*) occurs in all of the progeny of these five crosses but only because one of two parents carried this allele pair or because one parent carried allele (*U*) and the other carried allele (*u*).

Cross (1) typifies events in self-fertilising organisms but is not typical of sexual reproduction in other organisms (cf Figure 2 in reference [1]). Male and female parents that are *identically *heterozygous at any locus must be rare. Crosses (2)-(5) between two heterozygous parents will produce, under the circumstances noted above, truly homozygous allele pairs (such as *UU *and *uu*) but they will also produce, on average, three different heterozygotes among their progeny (four heterozygotes in the fifth cross).

The consequences are then as follows: From each locus in a diploid cell that expresses catalytic polypeptides, allozymes (pairs of enzymes) will be expressed; one from the gamete donated by the male parent the other from the gamete donated by the female parent. For simplicity, it will be assumed here that the combined allozyme activity at each catalytic locus in the metabolic pathways of the cell is the sum of the activities the two allozymes at each such locus.

The traditional allele series (*UU *+ 2*Uu *+ *uu*) in a diploid will then generate the enzyme series (*EE *+ 2*Ee *+ *ee*) at one metabolic locus in different, genetically related, individuals. This enzyme series provides two extreme combined allozyme activities, namely 100% (*EE*) and 0% (*ee*). There are no allele pairs at this locus that could provide <0% or >100% enzyme activity. All other allele pairs, e.g. (*UU*^†^), (*U*^†^*u*), (*U*^†^u*), (Uu*), (uu*), would provide combined allozyme activities that lie between the 100% and 0% values just described. Only if *(u) *happens to be a null mutant, will the heterozyote (*Uu*) express a single enzyme activity (*v*) equal to 50% of the maximum available from (*UU*). Only in this circumstance will the allele pair (*uu*) express two inactive polypeptides; the enzyme activity will then be zero at a metabolic locus and a "metabolic block" will occur at that locus.

Assembling the data from, for example, the second and third of the three hypothetical crosses between the genetically related parents described above gives an allele series (*UU*, *UU*^†^, *U*^†^*u*, *Uu*, Uu*, uu*, uu). They would contribute seven different allozyme pairs (*EE*, *EE*^†^, *E*^†^*e*, *Ee*, Ee*, ee*, *ee*) at one metabolic locus and seven different, single, enzyme activities (*v*), one from each pair of allozymes. Given a range of enzyme activities in excess of the traditional three, a sufficient number of co-ordinates will be available to establish a continuously non-additive plot of the response of one defined flux (*J*) against changes in enzyme activity (*v*) at one metabolic locus in genetically related cells or organisms (Figures 2, 3). There is no guarantee that all of these mutants will be generated in every case but since (*U*^†^) and (u*) each represent only one of several possible mutations of allele (*U*), we may be reasonably confident of observing traits expressed from allele pairs in addition to, or instead of, those expressed from the two traditional mutant pairs (*Uu*) and (*uu*). Assembling sets of enzyme activity and flux (or metabolite concentration) data from the progeny of different but genetically related parents then creates the non-linear flux response plots illustrated in Figures 2 and 3. All plotting points in the idealised Figures 2 and 3 should be regarded as tokens for the experimental plots published earlier [3].

This simple explanation for the occurrence of more than three co-ordinates for a plot of flux response against changes in enzyme activity (or gene dose) means that it is no longer acceptable to base arguments and conclusions on the assumed presence of only one heterozgote (*Uu*) in a diploid allele series at a locus, and on only one corresponding hybrid trait. Furthermore, statements that all heterozygotes express 50% (and only 50%) of the phenotype expressed from the homozygous wild-type are based on the false idea that the mutant allele (*u*) always produces a totally inactive enzyme. Figures 1a,1b,1c,1d,1e,1f,1g,1h of Reference [3] depended upon the availability of 5, 6 or 7 plotting points relating the flux response to experimentally determined changes in enzyme activity (effectively to changes in allele constitution at a locus). In addition to the traditional heterozygote (*Uu*), there must be a number of heterozygotes (e.g. *UU*^†^, *U*^†^*u*, Uu*, uu*), and a corresponding a range of enzyme activities (*v*), that account for the response of a flux (*J*) to a change in enzyme activity at one metabolic locus (Figures 1, 2, 3). In Figure 2, some of these additional heterozygotes will establish the asymptote of the flux response plot. The trait expressed from any such heterozygote would be indistinguishable from the trait expressed from the normal allele pair (*UU*); they could have accounted for the occurrence of Mendel's hybrids (*Aa*) which appeared to display only the dominant trait (*A*). This is further evidence that the traditional treatment of elementary Mendelian genetics is inadequate and misleading [1].

## 3. Quantifiable differences between any two forms of a trait

Differences in traits are generally and usefully described by qualitative terms:

hirsute/bald; red flowers/white flowers; lithe/obese; muscular/"skinny"; slow/fleet; albino/black. Such descriptive terms do, however, disguise the obvious fact these apparently qualitative differences in outward appearance are based on quantitative differences in the concentrations of molecular products that contribute to the outward appearance or function of a cell or organism.

These comments apply to the apparently qualitative differences examined by Mendel (Table 1 in reference [1]) and to those traits forms typified by a trait series (*A *+ 2*H *+ *a*) where (*A*) indicates the dominant trait form, (*a*) the recessive trait form and (*H*) a hybrid trait that may be indistinguishable (Figure 2) from the dominant traits (*A*) or distinguishable (Figure 3) from the dominant trait (*B*).

It should not therefore be supposed that the paper by Kacser and Burns [3] provided an explanation only for the occurrence of qualitative differences between any two traits. On the contrary, a continuously variable response of each of several defined fluxes was brought about when mutations of alleles at one locus changed the activity of one enzyme in a metabolic pathway (or when changes in gene dose changed the concentration and thus the activity of one enzyme in a metabolic pathway).

The flux responses were labelled "Flux to arginine", "Flux to biomass", "Flux to melanin", "Flux to products", "Flux to DNA repair" (Figure 1 in reference [3]). The molecular *compositions *of "arginine", "biomass", "melanin", and "products" (of ethanol metabolism) were not changed. Their *concentrations *were changed as graded mutations at a gene locus caused graded changes in one enzyme activity in those pathways that created arginine, biomass, melanin, or the products (of ethanol metabolism). Similarly, a change in the "flux to DNA repair" was achieved by graded increases in the dose of the gene specifying the synthesis of the "repair enzyme" that excises covalently-linked adjacent thymines in DNA and allows incorporation of thymidine in place of the excised pyrimidines. This "repair enzyme" activity is absent in *Xeroderma pigmentosum *patients.

Additional examples of quantitative changes in the concentration of molecular components of a trait will be found in other publications [5-11]. None of these changes provide any justification for representing a trait by twinned letters, e.g. (*AA*) or (*aa*). The single letters (*A*) and (*a*) stood for qualitative differences in trait form in Mendel's work; they stand equally well for quantitative changes in a trait in modern work. The non-linear response plots of Kacser and Burns [3] apply to quantitative and to apparently qualitative changes in the phenotype that arise from mutations of any one enzyme at a metabolic locus in a biochemical pathway.

## 4. Implications of the systemic approach of Kacser and Burns [3]

Figure 2 shows the response of the phenotype to changes in enzyme activity at a metabolic locus or to changes in gene dose at the corresponding gene locus. It follows, if the response plot takes this form, that *increasing *the dose of this particular gene in a wild-type haploid cell (or the dose of the normal homozygous alleles in a wild-type diploid or polyploid cell) is unlikely to produce a detectable change in the phenotype (e.g. an increase in the concentration of the trait component produced by a metabolic pathway; or a change in cell function associated with that pathway). It was demonstrated that it was necessary, under these circumstances, to increase concurrently the gene dose at each of no fewer than five loci if significant increases in the flux (and in the concentration of metabolic product) was to be achieved [5]. The systemic approach to a rational explanation of the origins of dominant and recessive traits [3] has obvious implications for biotechnologists.

Figure 2 (representing several plots in Reference [3]) also suggests that somatic recessive conditions (in contrast to so-called dominant conditions) could be ameliorated by partial gene replacement therapy. Experiments in the cystic fibrosis mouse model support this suggestion [7]; they show that the systemic approach to the origins of dominant and recessive traits has implications for medical genetics.

It was pointed out (section 2.6) that substantial *decreases *in the dose of normal alleles at any one locus (or in the enzyme activity at the corresponding metabolic locus) may not elicit detectable changes in the trait(s) of the cell. In other words, given a response plot approximating to that shown in Figure 2, traits – including associated cell functions – are inherently buffered against substantial increases or decreases in the dose of any one gene, or against substantial changes in enzyme activity at the corresponding metabolic locus. This appears to be the probable origin of the so-called "robustness" or buffering of chemotaxis against changes in enzyme kinetic constants [12-15].

This proposed explanation for metabolic buffering is quite general; it does not depend on the particular kinetic mechanisms that have been suggested to account for this buffering [12]; it also suggests that there is no need to postulate the presence of diagnostic "biological circuits" as the source of this buffering of the phenotype against mutations at a single locus.

Attempts to improve the concentration of metabolic products by increasing the gene dose at one locus above that available in the wild-type or normal cell could be successful, at least to some self-limiting extent, if a response plot like Figure 3 applies. Induced synthesis of one membrane-located enzyme activity to between 20% and 600% of wild-type activity illustrates the possibility [8]. In this instance, plots like Figure 3 applied only to changes in the uptake and phosphorylation of α-methyl glucoside; changes in growth rates and glucose oxidation gave response plots like Figure 2. The explanation for the difference may lie in the suggestion [3] that shorter pathways will yield response plots like Figure 3, while the longer the pathway, the more likely is it that markedly asymmetric plots like Figure 2 will be observed.

## 5. Expansions of the present treatment

### 5.1. Why mutating one enzyme in a metabolic pathway may alter more than one trait; and mutating more than one enzyme may annul these changes in more than one trait

If the explanation for the origin of dominant and recessive traits depends on realising that fluxing metabolic pathways generate the molecular components of all traits, and that mutating any one enzyme in these pathways alters the flux and the concentrations of those normal metabolic products that are molecular components of a trait, other genetic phenomena could perhaps also be explained. Only two of the thirteen texts surveyed [1] gave a definition, in their glossaries, of pleiotropy and epistasis. Both agreed that pleiotropy was a phenomenon where a change at one gene locus brought about a change in more than one trait. Both attributed epistasis to an interaction between genes or their alleles. Neither of these descriptions of pleiotropy and epistasis is particularly revealing.

**Figure 4:**
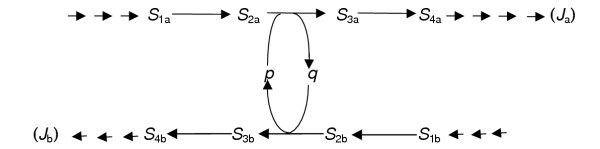
Accounting for the occurrence of pleiotropy. One unbranched pathway is coupled to another by a conserved metabolite pair *p *and *q*. Such coupling is not uncommon in cellular systems and is one source of pleiotropy. Mutation of any one enzyme in one pathway will affect both fluxes (*J*_a _and *J*_b_) to a trait component and the concentrations of those trait components. See also Figure 5. Figure 4, like Figure 1, illustrates the need to adopt a systemic approach in attempts to understand the responses of a metabolising system to changes in any enzyme activity brought about by mutation.

The following account, like those preceding it, does not depend on the fiction that all mutations generate inactive enzymes. Figure 1 is elaborated as shown in Figure 4. One pathway, like that shown in Figure 1, is now coupled to another analogous pathway by the conserved metabolite pair (*p*, *q*). The sum of the concentrations of (*p*) and (*q*) is constant (is conserved) but the ratio of the two concentrations (*p*/*q*) is a free variable. All the characteristics of the metabolic system in Figure 1 (Section 2), apply to each of the two fluxing pathways in Figure 4. Claims in the biochemical literature in the past that changes in the ratio (*p/q*) controlled metabolic fluxes were and remain untenable; one variable of a system cannot be said to control another variable of the system.

**Figure 5:**
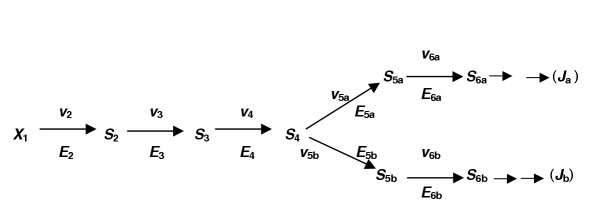
Accounting for the occurrence of pleiotropy and epistasis. Mutation of any one of enzymes *E*_2_, *E*_3_, *E*_4 _would affect both fluxes *J*_a _and *J*_b _to separate trait components. Mutation of any one of enzymes *E*_5a_, *E*_6a_, etc would decrease flux *J*_a _to a trait component but increase *J*_b _to another trait component; the concentrations of trait components in pathway *J*_a _would decrease, those in pathway *J*_b _would increase. Epistasis would occur if a subsequent mutation occurred in any one of enzymes *E*_5b_, *E*_6b _etc. A branched metabolic pathway is thus a potential source of pleiotropy and epistasis; see the text for further discussion. This diagram, like that in Figure 4, emphasises the importance of adopting a systemic approach in understanding the potential effect, on a trait or traits, of a mutation in any one enzyme in enzyme-catalysed systems.

Figure 1 may also be elaborated as shown in Figure 5. An input flux from *X*_1 _to *S*_4 _divides into two output fluxes [16]. Of the input flux, a proportion (α) enters one of the two output fluxes (*J*_a_) and a proportion (1-α) enters the other output flux (*J*_b_). The magnitude of (α) is determined by the magnitudes of the activities of all the enzymes of the metabolic system; (α) is a systemic characteristic [17]. Again, all the characteristics of the model metabolic system in Figure 1 (Section 2), apply to each of the two pathways that generate fluxes *J*_a _and *J*_b _shown in Figure 5.

### 5.2. The origin of pleiotropy explained

It will be obvious that a mutation of any one enzyme in either of the two pathways of Figure 4 will cause changes in the fluxes through both of the coupled pathways (and the concentrations of metabolites in both pathways). Similarly, a mutation in any one enzyme of the input flux of Figure 5 will affect the concentrations of metabolites in both output fluxes *J*_a _and *J*_b_. Pleiotropy (a change in more than one trait as a consequence of a single mutation), when it is detected, is thus seen to depend on mutating an enzyme within a metabolic pathway, on the consequential changes in metabolite concentrations, and on the structure and interdependence of biochemical pathways. Only if one of the enzymes in the input pathway shows zero activity will both output fluxes (*J*_a _and *J*_b_) cease (Figure 5).

### 5.3. The origin of epistasis explained

Given a steady input flux from *X*_1 _to *S*_4 _(Figure 5), a mutation of one of the enzymes (*E*_5a_, *E*_6a _or any other enzyme in this output limb) would decrease flux *J*_a _and increase flux *J*_b_. The concentrations of metabolites in pathway *J*_a _would decrease and those in pathway *J*_b _would increase, a further example of a pleiotropic response to a single mutation. But suppose that, following the mutation of *E*_5a_, a mutation occurred in *E*_6b _or any other enzyme in this alternative output limb. Clearly, the effect of the first mutation on the cell characteristics would be at least partly nullified by the second mutation – a phenomenon known as epistasis and sometimes attributed in genetic texts to an interaction between genes but shown here to depend on mutations of one or more enzymes, and on the structure and interdependence of metabolic pathways. Only if the activity of one of the enzymes in one of the two output pathways is diminished to zero by mutation, will the products of that output limb downstream from the mutation be lost.

If the fluxes proceeded in the opposite direction to that shown in Figure 5 (so that two pathways merged into one), mutation of an enzyme in one of the input fluxes followed by a mutation of an enzyme in the other input pathway could again elicit epistatic responses in the system.

### 5.4. Are pleiotropy and epistasis always detectable?

Particular but common metabolic structures (Figures 4, 5) provide the potential for pleiotropy and epistasis; i.e. changes in concentrations of normal metabolites when an enzyme is mutated within a metabolic pathway. Whether pleiotropy or epistasis is detected, or not, will depend on the severity of the mutation and on the nature of the flux response plots (Figures 2, 3) as demonstrated in section 2.

### 5.5. Biochemistry and genetics are not separable topics

Beadle and Tatum [18] isolated a series of mutants of *Neurospora crassa *and tested their ability to grow on basal medium or on basal medium supplemented with different metabolites or cofactors. Wild-type *Neurospora crassa *grew on basal medium. Different isolated mutants would grow only if the basal medium was supplemented with the specific product of an enzyme rendered partially or fully inactive in one of the mutants. These brilliant observations led to the paradigm "one gene, one function" [19,20], later to "one gene, one enzyme". These observations [18] made explicit what was implied by the observations of Garrod [21-24]] on inborn errors of metabolism namely: metabolism is catalysed by a sequence (or system) of different enzymes; and a sufficient decrease (by mutation) in the activity of any one enzyme may cause a change in the trait(s) or characteristic(s) of the system (e.g. the ability to grow, to accumulate cell mass [18]).

Beadle [20] expressed surprise that Garrod's work had received so little attention. He wrote: "It is a fact both of interest and historical importance that for many years Garrod's book had little influence on genetics. It was widely known and cited by biochemists, and many geneticists in the first two decades of the century knew of it and the cases so beautifully described in it. Yet in the standard textbooks written in the twenties and thirties - - - - few mention its cases or even give a reference to it. I have often wondered why this was so. I suppose most geneticists were not yet inclined to think of hereditary traits in chemical terms. Certainly, biochemists with a few notable exceptions such as the Onslows, Gortner and Haldane were not keenly aware of the intimate way in which genes direct the reactions of living systems that were the subject of their science."

This lack of attention to the implications of Garrod's work is all the more surprising when it is recalled that Bateson [[25], p.133] pointed out that alkaptonuria (a change in concentration of the normal metabolite, homogentisic acid, and one of Garrod's inborn errors of metabolism) was an example of a Mendelian recessive trait or character; see also [[26], p.19]. In other words, some important aspects of genetics depended on recognising the role of changes in an enzyme activity, within a metabolic system, in effecting a change in a trait.

The aphorism "one gene, one enzyme" was refined to "one allele, one polypeptide" after the elucidation of the structure of DNA [27,28] and the rapid advances made in the next 10 or 15 years in elucidating the mechanisms of expression of diploid alleles as pairs of polypeptides or proteins [29-32]] most of which are enzymes [3,4]. These more recent discoveries (Figure 6) emphasise what was implied by the work of Beadle and Tatum [18]: the molecular components of dominant and recessive traits or characteristics, in all biological forms, are generated by fluxing metabolic pathways catalysed by sequences or systems of enzymes. Dominant and recessive traits are not the direct product of the expression of alleles as suggested by the currently favoured explanation of Mendel's observations (Figure 2 in Reference [1]); they are produced *indirectly *by a system of enzymes (Figures 1, 4, 5, 6).

Figure 6 depicts the direct relationship between any one gene (g1, g2, g3, g4) and the synthesis of individual polypeptides (P_1_, P_2_, P_3_, P_4_) most of which, but not all, are enzymes (*E*_1_, *E*_2_, *E*_3_, *E*_4_). All polypeptides, catalytic and non-catalytic, are synthesised in this way.

*X*_1_, *X*_2 _and *X*_3 _in Figure 6 are immediately identified as extracellular parameters of a cell system. *X*_3 _stands for those substrates that lead, through a series of enzyme-catalysed reactions, to the synthesis of nucleoside triphosphates (NTPs) and their subsequent incorporation into mRNA. Note that mRNA is a terminal product of this pathway. It is a coding entity, a proxy for DNA. Each mRNA specifies the order of incorporation of individual amino acids into a polypeptide, but no individual mRNA molecule participates as a substrate in the subsequent steps of the catalysed formation of a polypeptide. The control of the overall expression of a gene as a polypeptide is therefore necessarily treated in Metabolic Control Analysis as a cascade of two fluxing metabolic pathways, one that starts at *X*_3_, the other that starts at *X*_2 _[33].

*X*_2 _stands for those extracellular substrates that lead, through a series of enzyme-catalysed reactions, to the synthesis *de novo *of amino acids (AAs) and their subsequent incorporation, along with any existing amino acids, into a polypeptide (P). In a haploid cell, one polypeptide is synthesised from each gene locus. In a diploid, one polypeptide is synthesised from each of two alleles at a gene locus. If these pairs of polypeptides are catalytically active, each enzyme in a diploid cell (*E*_1_, *E*_2_, *E*_3_, etc) consists of a pair of allozymes, one of each pair specified by the allele derived from the male parent, the other specified by allele derived from the female parent. Each pair of allozymes, whether normal or mutated, exhibits only one measurable activity (*v*) at a catalytic locus in a metabolic pathway. If the pairs of polypeptides (P) synthesised by a diploid cell are *not *catalytically active they will not, of course, play a direct role in catalysing a metabolic pathway. They may have other important functions (e.g. as hormones) and may be components of traits.

*X*_1 _stands for all those initial extracellular substrates feeding the matrix of inter-dependent biochemical pathways that typify all functioning cells. It is these pathways that generate the non-protein, non-polyribonucleotide, molecular products of all cell traits.

Each of these three major fluxing pathways (Figure 6) is catalysed by a succession of enzyme-catalysed reactions as shown in Figure 1. The flux through any one of these pathways will respond to a mutation of any one enzyme in the pathway as shown in Figures 2, 3; any change in these fluxes could change the concentrations of the intermediate metabolites or the final product (section 2.3); but, provided mutations do not alter the specificity of an enzyme, they will not change the existing molecular structure or composition of these metabolites.

Most attention is concentrated on the pathway initiated by *X*_1 _for the simple reason that this pathway stands for all the matrix of interdependent biochemical fluxes that generate such a wide range of the non-protein (and non-polyribonucleotide) molecular components of cell traits (e.g. skin pigments, membrane lipids, chlorophyll, xanthocyanins, non-peptide hormones, neural transmitters, chitin, serum cholesterol, peptidoglycans, etc, etc).

If any one of the three major pathways shown in Figure 6 is coupled to another pathway (Figure 4) or contains a branch (Figure 5) there will be, potentially detectable, pleiotropic and epistatic responses to mutations of any of the pathway enzymes (section 5.3). Such pathway coupling and branching is a common feature of the pathways that start with one of the extracellular substrates typified by *X*_1_.

If the implications of the work of Beadle and Tatum [18] were not fully realised at the time, Figure 6 might have suggested that a fresh approach to an understanding of the origins of dominant and recessive traits was needed. The currently favoured explanation for Mendel's findings ([1], Figure 2) does not take account of the biochemical pathways of the synthesis of enzymes (Figure 6) established 30–40 years ago, does not acknowledge that the molecular components of all traits are synthesised by systems of enzymes, does not take account of the change in concentration of molecular components of traits when any one enzyme is mutated, and fails to distinguish the system parameters (alleles) from the system variables (traits).

Note that changes in the concentrations of external metabolites (whether they are substrates like *X*_1_, *X*_2_, *X*_3_in Figure 6, or extracellular inhibitors or activators of intracellular enzymes) may effect changes in intracellular metabolism and consequently modify the effects of a mutation. This topic is not immediately relevant in the present article but is a notable feature of Metabolic Control Analysis. Descriptions of the role of the Combined Response Coefficient (*R*) in permitting extracellular effectors to modulate intracellular metabolism (and thus the effects of a mutation) will be found elsewhere [11,34-36].

If pleiotropic and epistatic responses to a mutation are as common as is suggested (sections 5.1–5.4), the question then arises: how do we account for Mendelian segregation of traits during sexual reproduction? The answer lies in the fact that a mutation at a biochemical locus, within the matrix of interdependent pathways, has its most obvious effect on the most closely associated pathways. Distant pathways (on the scale of cellular dimensions) will be less obviously affected. Kacser and Burns (Reference [3], p.649) pointed out that "This apparent independence of most characters makes simple Mendelian genetics possible, but conceals the fact that there is universal pleiotropy. All characters should be viewed as 'quantitative' since, in principle, variation anywhere in the genome affects every character." Section 3 in the present article emphasised the importance of quantitative changes in cell traits. The considerations in this paragraph are germane to the apparent absence of a detectable change of phenotype in some so-called 'knock-out' experiments.

## 6. Conditions that must be met to explain dominance and recessivity

The explanation advocated in this article for the origins of dominant and recessive traits from normal and mutant alleles in a diploid is based on:

(i) An obligatory distinction, by notation and nomenclature, between the variables (traits) and the parameters (alleles and enzymes) of genetic/biochemical systems.

(ii) The contention that the molecular components of all traits are the products of fluxing metabolic systems (Figures 1, 4, 5, 6).

(iii) Experimental evidence for an inevitable non-linear response of a flux (through a metabolic system of enzymes) to graded changes in the activity of any one of those enzymes [3], evidence that is supported by a number of independent observations [5-11].

(iv) A demonstration that dominant and recessive traits arise from changes in the concentration of the normal molecular components of a defined trait.

(v) The argument that changes in concentration of a trait component may nevertheless be revealed as a qualitative change in that trait.

(vi) A demonstration that both alleles (normal or mutant) at a locus in a diploid are generally expressed. If the normal allele expresses a catalytically active polypeptide, many mutants of this allele will express an enzyme with lower activity; a mutated enzyme with zero activity is an extreme case.

(vii) The demonstration that an explanation of Mendel's observations cannot be based on an allele series containing only three terms (e.g. *uu*, 2*uU*, *UU*) one of which is a unique heterozygote (*uU*).

(viii) A demonstration that dominant and recessive traits cannot be generated by those polypeptides that are not enzymes embedded in a system of enzymes.

(ix) Rejection of the unjustified traditional claim that a hybrid (*H*) expresses a dominant trait (*A*) because the (allegedly) recessive allele (*u*) in a heterozygote (*Uu*) is always completely ineffective or because the allegedly dominant allele (*U*) suppresses the allegedly recessive allele (*u*) in the heterozygote [1].

(x) Rejection of the traditional, unsubstantiated and implausible claim that one so-called dominant allele in a heterozygote is as effective as two such alleles in the wild-type cell [1].

It was also shown that pleiotropy and epistasis can be explained by taking a similar system approach to that used in explaining the origin of dominant and recessive traits.

It is then apparent that, to account rationally for Mendel's observations of dominant and recessive traits, a minimum of four conditions must be met.

(i) Alleles must be distinguished by notation, nomenclature and concept from traits; functions of components of the genotype must be distinguished from properties of components of the phenotype. Traits alone may be dominant or recessive.

(ii) Alleles cannot be called "dominant" or "recessive". (When alleles are so called, the flaws present in the current attempts to explain Mendel's observations will inevitably re-appear [1]).

(iii) It must be shown how dominant traits become distinguishable from recessive traits in the same cell or organism (Figure 2, 3).

(iv) It must be shown how a hybrid trait sometimes becomes indistinguishable from the dominant trait and sometimes does not (Figures 2, 3). The first circumstance will account for Mendel's 3(dominant):1(recessive) trait ratio; the second for exceptions to this ratio.

If all four conditions are be met; the first two conditions must first be met. The treatment given in sections 2–5 meets each of these requirements.

## 7. Conclusions

Kacser and Burns [3] provided the basis for a rational explanation for the *origin *of dominant and recessive traits that arose from mutations of alleles at any one gene locus in a diploid or polyploid cell (sections 2.3, 2.4). Inherent in this explanation, as set out above (sections 2.5, 2.6), are further explanations for the occurrence of the 3(dominant):1(recessive) trait ratio in some situations in a diploid (Figure 2), for the absence of this trait ratio from other situations (Figure 3), for the absence of dominant and recessive traits in yet others and for the appearance of a blend of parental traits in some heterozygotes. These five demonstrations are internally consistent. In contrast to the currently favoured attempt to explain Mendel's results [1], no arbitrary assumptions are introduced (section 2.8) to explain how heterozygous allele pairs (e.g. *UU*^†^, *U*^†^*u*, Uu*, uu*) may produce a trait that is indistinguishable from the trait expressed from the "homozygous" allele pairs (*UU*).

In other words, provided:

(a) all current misrepresentations of Mendel's paper [1] are first discarded,

(b) alleles are distinguished by notation and nomenclature from the traits they specify,

(c) alleles are regarded as normal or mutant (but not dominant or recessive), it is possible to provide a rational and internally consistent explanation for the origin of Mendel's dominant and recessive traits, for the occurrence of his 3:1 trait ratio, and for exceptions to these observations noted by later investigators. The same systemic approach is applicable to current problems in biotechnology and medical genetics (section 4). It also explains the origins of pleiotropy and epistasis (section 5); and challenges the assumption that a mutation in a non-catalytic protein provides an example of Mendel's dominant and recessive traits [1].

Mendel found, by experiment, that the proportions of plant forms in each of his F2 populations was represented by (*A *+ 2*Aa *+ *a*). In the present paper these proportions have been written as (*A *+ 2*H *+ *a*). If the symbol (*H*) for a hybrid in Figure 2 is replaced mentally and temporarily by (*Aa*), it will be clear why Mendel postulated that his hybrids (*Aa*) displayed trait (*A*) and not trait (*a*). If the same exercise is repeated in Figure 3 by replacing (*H*) temporarily by (*Bb*), it will be clear why Mendel observed an anomalous blending of flower colours in the hybrids when he crossed parental bean plants bearing different flower colours.

The treatment of elementary Mendelian genetics advocated here is based on the work of Kacser and Burns [3]. So far as the present author is aware, that paper has not been described by any student textbook of "classical" or "molecular" genetics published in the intervening 23 years. Orr [37] did not see the full significance of the Kacser and Burns paper [3]. Darden [[38], p. 72] declared that "(trying) to unravel the complex relations between mutant alleles and enzymes (Kacser and Burns, 1981) - - - is not a major research topic in genetics."

Several possible reasons for this failure to see the merits of the Kacser and Burns paper [3] may be worth consideration. They include:

(1) Persistent misrepresentations of Mendel's paper, and incorporation of these distortions into currently favoured explanations of Mendel's observations [1].

(2) A failure to recognise the consequences of not distinguishing between the function of the alleles and the properties of traits in attempting to explain Mendel's results. Normal and mutant alleles *specify *the kind (and order of incorporation) of amino acids into polypeptides (most but not all are enzymes). Dominance and recessivity are a reflection of changes in the *concentration(s) *of the molecular component(s) of a trait when an enzyme is mutated within a fluxing metabolic pathway.

(3) Tardy recognition of the need to adopt the systemic approach of Metabolic Control Analysis in explaining the response of the *variables *of a biological system to perturbations of the magnitude any one system *parameter*.

(4) A reluctance to accept a change in concepts even when currently accepted representations of Mendel's results are demonstrably untenable.

(5) Elucidation of the double helical structure of DNA (Figure 6) and all that followed in the next 10–15 years imposed profound changes on genetics but was not perhaps always taken into account.

(6) A determination in some quarters to regard genetics as an autonomous subject. It has been obvious at least since the work of Beadle and Tatum [18] that such claims cannot be sustained. Genetics is intimately related to, and in some respects dependent upon, biochemistry. The converse is equally true. Genetics and biochemistry are not separable topics in biology.

It is significant that Kacser & Burns were also one of two sets of authors who initiated the systemic approach to the control of metabolite concentrations and fluxes [39,40]. This approach was elaborated by the original authors and many others. For some accounts and reviews, see [11,36,41-44].

## 8. A correction

In an earlier paper [45] it was stated that Mendel had inferred the presence of segregating particles. These particulate determinants were then represented by (*A*) and (*a*). These statements are here formally withdrawn. They were consistent with textbook treatments of Mendelian genetics [1] but a subsequent reading of Mendel's original paper revealed that these statements, and others that occur frequently in the recent reviews of Mendel's paper and in current textbooks, were incorrect and misleading. A history of the misunderstandings and misrepresentations that have sustained the currently favoured depiction of Mendelian genetics [1] will be presented elsewhere. A paper setting out the concepts of parameters and variables will also be submitted.

## References

[B1] Porteous JW (2004). We still fail to account for Mendel's observations. Theor Biol Med Modelling.

[B2] Mendel G (1866). Versuche über Pflanzen-Hybriden. Verhandlungen des Naturforschenden Vereines in Brunn.

[B3] Kacser H, Burns JA (1981). The Molecular Basis of Dominance. Genetics.

[B4] Nirenberg MW (1963). The Genetic Code II. Sci Amer.

[B5] Niederberger P, Prasad R, Miozzari G, Kacser H (1992). A strategy for increasing an *in vivo *flux by genetic manipulations. The tryptophan system of yeast. Biochem J.

[B6] Dean AM, Dykhuisen DE, Hartl DL (1986). Fitness as a function of β-galactosidase activity in *Escherichia coli*. Genet Res.

[B7] Dorin JR, Farley R, Webb S, Smith SN, Farini E, Delaney SJ, Wainwright BJ, Alton EWFW, Porteous DJ (1996). A demonstration using mouse models that successful gene therapy for cystic fibrosis requires only partial gene correction. Gene Therapy.

[B8] Ruyter GJG, Postma PW, van Dam K (1991). Control of glucose metabolism by EnzymeII^Glc ^of the phosphoenolpyruvate-dependent phosphotransferase system in *Escherichia coli*. J Bact.

[B9] Letellier T, Heinrich R, Malgat M, Mazat J-P (1994). The kinetic basis of threshhold effects observed in mitochondrial diseases: a systemic approach. Biochem J.

[B10] Small JR, Kacser H (1993). Responses of metabolic systems to large changes in enzyme activities and effectors. 1. The linear treatment of unbranched chains. Eur J Biochem.

[B11] Fell DA (1997). Understanding the Control of Metabolism.

[B12] Barkai N, Leibler S (1997). Robustness in simple biochemical networks. Nature.

[B13] Hartwell L (1997). A robust view of biochemical pathways. Nature.

[B14] Cohen P Weathering the storm. New Scientist 2000 Online Conference Reports.

[B15] Ferber D (2000). The spice of life. NewScientist Online Conference Report.

[B16] Kacser H (1983). The control of enzyme systems in vivo: elasticity analysis of the steady state. Biochem Soc Trans.

[B17] Fell DA, Sauro JM (1985). Metabolic control analysis. Additional relationships between elasticities and control coefficients. Eur J Biochem.

[B18] Beadle GW, Tatum EL (1941). Genetic control of biochemical reactions in Neurospora. Proc Natl Acad Sci USA.

[B19] Beadle GW (1945). Genetics and metabolism in Neurospora. Physiol Rev.

[B20] Beadle GW, Dunn LC (1951). Chemical genetics. Genetics in the 20th Century.

[B21] Garrod AE (1901). About Alkaptonuria. Lancet.

[B22] Garrod AE (1902). The incidence of Alkaptonuria. Lancet.

[B23] Garrod AE (1908). Croonian Lectures to the Royal Society of Physicians: Inborn Errors of Metabolism. Lancet.

[B24] Garrod AE Inborn Errors of Metabolism (a revision of the Croonian Lectures, 1909).

[B25] Bateson W (1902). Report of the Evolution Committee of the Royal Society.

[B26] Harris H (1963). Garrod's Inborn Errors of Metabolism.

[B27] Watson JD, Crick FHC (1953). Molecular structure of the nucleic acids. A structure for deoxyribonucleic acid. Nature.

[B28] Watson JD, Crick FHC (1953). Genetical implications of the structure of deoxyribonucleic acid. Nature.

[B29] Crick FHC (1958). On protein synthesis. Symp Soc Exp Biol.

[B30] Crick FHC (1966). The Genetic Code III. Sci Amer.

[B31] Taylor JH (1967). Molecular Genetics II.

[B32] Yanofsky C (1967). Gene Structure and Protein Structure. Sci Amer.

[B33] Westerhoff HV, Koster JG, van Workum M, Rudd KE, Cornish-Bowden A, Cárdenas ML (1990). On the control of gene expression. Control of Metabolic Processes.

[B34] Kacser H, Porteous JW (1987). Control of Metabolism: What do we have to measure?. Trends Biochem Sci.

[B35] Porteous JW, Cornish-Bowden A, Cárdenas ML (1990). A theory that works. Control of Metabolic Processes.

[B36] Cornish-Bowden A (1995). Fundamentals of enzyme kinetics.

[B37] Orr HA (1991). A test of Fisher's theory of dominance. Proc Natl Acad Sci USA.

[B38] Darden L (1991). Theory change in science.

[B39] Heinrich R, Rapoport TA (1973). Linear theory of enzymatic chains; its application for the analysis of the cross-over theorem and the glycolysis of human erythrocytes. Acta Biol Med Germanica.

[B40] Kacser H, Burns JA (1973). The control of flux. Symp Soc Exp Biol.

[B41] Fell DA (1992). Metabolic Control Analysis: a survey of its theoretical and experimental development. Biochem J.

[B42] Kell DB, Westerhoff HV (1986). Metabolic control theory; its role in microbiology and biotechnology. FEMS Microbiol Rev.

[B43] Teusink B, Baganz F, Westerhoff HV, Oliver SG (1998). Metabolic Control Analysis as a tool in the elucidation of novel genes. Methods Microbiol.

[B44] Wildermuth MC (2000). Metabolic control analysis: biological applications and insights. Genome Biology.

[B45] Porteous JW (1996). Dominance – One hundred and fifteen years after Mendel's paper. J Theor Biol.

